# P10-06 Home-based videoconference vs. face to face physical training in healthy older adults

**DOI:** 10.1093/eurpub/ckac095.145

**Published:** 2022-08-29

**Authors:** Antoine Langeard, Lucile Bigot, Nicola Maffiuletti, Sébastien Moussay, Antoine Gauthier, Gaëlle Quarck

**Affiliations:** 1 COMETE U1075, INSERM/UNICAEN, Caen, France; 2 MOOVEN, Caen, France; 3 Schulthess Clinic, Zurich, Switzerland; 4 BodyCap, Hérouville Saint Clair, France

**Keywords:** telecare, physical training, body composition, cardiorespiratory fitness, muscle strength

## Abstract

**Background:**

Older adults often fail to reach the recommended amount of physical activity to prevent the age-related decline in metabolic, cardiorespiratory, and muscular function. Effective home-based physical training programs could neutralize barriers preventing older adults from being active, and administration/supervision through videoconference may be an optimal solution. The present randomized controlled trial aimed to test the non-inferiority of training program administered through videoconference against the same program administered face-to-face in healthy older adults.

**Methods:**

Participants were randomized in a no-training control group (n = 13), a face-to-face training group (n = 15), and a videoconference training group (n = 13). The intervention groups completed the same home-based, structured, progressive and combined training program for 16 weeks, 1-hour twice a week. Pre-intervention and post-intervention evaluations included body composition, cardiorespiratory fitness and muscle function measures. The non-inferiority margin was calculated by comparing the face-to-face training group to the no-training control group.

**Results:**

Non-inferiority of videoconferencing against face-to-face training was observed for changes in body weight (p>.01), fat mass (p=.015), maximal aerobic power (p=.013), maximal heart rate (p=.034), maximal oxygen consumption (p>.01), knee extension strength (p=.044) and lower limb power (p=.019), but not for muscle mass (p=.067), handgrip strength (p=.171), trunk extension strength (p=.241) and knee flexion strength (p=.462).

**Conclusion:**

A training program administered through videoconferencing was not inferior to the same program administered face-to-face for reducing body weight and fat mass, and for improving maximal aerobic power and oxygen consumption as well as lower limb power and knee extension strength in healthy elderly subjects. However, videoconferencing training was not as effective as face-to-face training for improving handgrip, trunk extension and knee flexion isometric strength, possibly because of a higher motivation related to the physical presence of the trainer in the face-to-face training group.

